# Pluripotent stem cells in neuropsychiatric disorders

**DOI:** 10.1038/mp.2017.40

**Published:** 2017-03-21

**Authors:** M A Soliman, F Aboharb, N Zeltner, L Studer

**Affiliations:** 1 Weill Cornell Medical College, Cornell University, New York, NY, USA; 2 Developmental Biology and Center of Stem Cell Biology, Sloan-Kettering Cancer Center, New York, NY, USA; 3 Rockefeller University, New York, NY, USA

## Abstract

Neuropsychiatric disorders place an enormous medical burden on patients across all social and economic ranks. The current understanding of the molecular and cellular causes of neuropsychiatric disease remains limited, which leads to a lack of targeted therapies. Human-induced pluripotent stem cell (iPSC) technology offers a novel platform for modeling the genetic contribution to mental disorders and yields access to patient-specific cells for drug discovery and personalized medicine. Here, we review recent progress in using iPSC technology to model and potentially treat neuropsychiatric disorders by focusing on the most prevalent conditions in psychiatry, including depression, anxiety disorders, bipolar disorder and schizophrenia.

## Introduction

### Global burden of psychiatric disorders

**Figure 1:**
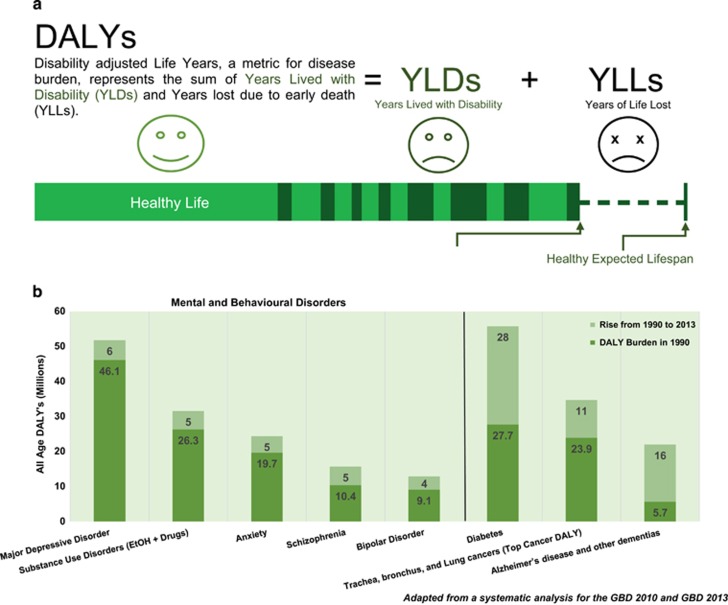
(**a**) Graphic representation of disability-adjusted life years (DALYs), years lived with disability (YLDs) and years of life lost (YLLs). (**b**) Graph representing the DALY burden of leading neuropsychiatric disorders, as well as comparable non-neuropsychiatric diseases.

Neuropsychiatric illnesses greatly burden the health care system, economy and wellbeing of affected patients and their dependents worldwide. The magnitude of this problem is illustrated by several facts. First, when assessed in disability-adjusted life years (DALYs)—a measure of the number of years lost because of poor health, disability and early death—mental illness and substance use disorders accounted for a total of 173.1 million DALYs or roughly 7.1% of total disease burden worldwide (Figure 1a).^1^ In the United States, major depression ranked 5th and anxiety ranked 13th out of 291 medical conditions contributing to DALY burden.^2^ Globally, major depressive disorder (MDD) ranked 5th among the top 10 causes for disability in developed countries.^1^ Second, the global burden attributable to mental diseases has been steadily rising in recent years. Between 1990 and 2010, the burden of neuropsychiatric and substance use disorders has increased by nearly 22% (Figure 1b).^3^ Recently, Vigo *et al.*^4^ compellingly argued that the global burden of mental illness is likely underestimated by more than one-third, suggesting that the effect of psychiatric disease may be even more dramatic than currently estimated. Finally, mental illness also leads to a significant economic impact estimated to be equivalent to the cost of cancer care.^5^ Serious mental illness was estimated to be associated with a loss of $193.2 billion in personal earnings across the United States.^6^

Despite the fact that many epidemiologic studies on neuropsychiatric diseases are conducted in the developed world, mental health represents a major problem globally. Of the 188 countries examined in the Global Burden of Disease Study 2013, depression was in the top 10 causes of DALYs for 130 countries (69%).^1^ These figures are worrisome given that outside of the western world, additional factors further complicate patient care. For example, psychiatrists and persons trained to treat mental illness are exceptionally scarce in many poor countries.^7^ It is estimated that 75% of mental health patients in low-income countries do not have access to care.^8^ Moreover, social and cultural factors lead to a stigma surrounding mental disorders, which deters patients from seeking appropriate treatment.^9^ Taken together, this grim picture highlights the tremendous need for an in-depth understanding of and effective treatments for mental disorders. It also raises the important question: Where does research on neuropsychiatric illness currently stand?

## Understanding neuropsychiatric disease

### Challenges in research

Despite the great progress made in recent decades, our knowledge of the pathophysiology of many common neuropsychiatric disorders remains limited. The lag experienced in this domain compared with other medical fields is not without cause; several unique obstacles exist in the study of mental diseases. First, mental disorders represent dysfunction in the least understood organ of the human body: the brain. Second, our limited understanding for the genetic basis of mental illness suggests great complexity. Studies on twins repeatedly show that the genetic makeup of affected neuropsychiatric patients contributes to their disease.^10^ For example, heritability for bipolar disorder (BPD) and schizophrenia (SCZ) is estimated to be between 80 and 90%.^11, 12^ This degree of heritability is higher than that associated with breast cancer (30%),^13^ type II diabetes (50–70%)^14^ and hypertension (40–60%).^15^ Moreover, genome-wide association studies show that psychiatric diseases are generally polygenic, with several genetic variants contributing a fraction of the overall risk and phenotype.^16^ Further complicating matters, many of these genetic variants exist in non-coding regions with unknown functions.^16^ Notable exceptions include Rett and Fragile X Syndromes, which arise from defined monogenetic risk factors.^17^

Third, a paucity of model systems stifles the study of neuropsychiatric disorders. Despite the great utility of animal models in probing conserved neuronal molecular pathways and elucidating neuronal circuits, they fail to fully recapitulate any of the psychiatric disorders defined in the Diagnostic and Statistical Manual of Mental Disorders, Fifth Edition (DSM-5). The DSM-5-defined diseases also likely represent an assortment of more circuit-specific pathologies, each triggering a related set of symptoms. Therefore, it may not be feasible to capture a given neuropsychiatric disorder in a single model organism. The limited access to disease-relevant tissue and cell types in humans represents a major obstacle toward probing disease mechanisms. To gain greater insight at the molecular level, the scientific community uses patient biopsies, cell lines and post-mortem tissue. Each of these approaches remains suboptimal for studying neuropsychiatric diseases. Biopsies of brain tissue are highly invasive, ethically problematic to obtain and yield little material. Moreover, post-mitotic neurons cannot be expanded *in vitro*. Commonly used cell lines do not apply to the study of neuropsychatric diseases, and only a few immortalized brain-derived cell lines exist. Furthermore, the few relevant cell lines fail to fully recapitulate disease etiology or patient genetics. Currently, the best method of accessing patient-specific brain tissues is post-mortem sampling, which typically represents late stages of disease and offers little direct insight into the pathogenesis of disorders that often present early in life. In addition, these tissues rarely represent the natural course of disease progression because most patients receive some form of medical or psychological intervention after diagnosis. Layered on these confounding factors are the various comorbidities that accompany mental illness and variations in artifacts owing to different methods of fixation and storage of post-mortem tissues. Thus, advancing our understanding of neuropsychiatric disease will rely on technology that allows greater access to human neuronal cell types affected in mental illness and is capable of capturing patient genetic makeup. Such an advance would facilitate the study of neuropsychiatric disorders at a cellular and molecular level and the role of genetic variants in disease etiology. This will also provide a new platform for drug screening to identify new and improved therapies.

### Induced pluripotent stem cells: development backward

In 1998, Thomson *et al.*^18^ first established the technique of isolating human embryonic stem cells (hESCs) from blastocysts. When cultured, these cells can be maintained in an undifferentiated, pluripotent state indefinitely, enabling researchers to generate an unlimited number of cells. Furthermore, hESCs are pluripotent and thus can differentiate into any human tissue, including neurons and glia when guided through the appropriate developmental pathways. This is traditionally achieved through exposing hESCs to a combination of growth factors and modulators of specific signaling pathways. These protocols can generate progenitor cell types along the developmental spectrum and adult-like cell types of varying maturity.^19^ Hence, hESCs opened the door to investigating early human development and understanding molecular mechanisms driving cellular differentiation. However, the need to harvest these cells from human embryos generated considerable ethical controversy. This was resolved by the Nobel prize-winning discovery that adult somatic cells can be reprogrammed into cells with embryonic stem cell properties by the introduction of as few as four specific transcription factors.^20^ These cells were termed induced pluripotent stem cells (iPSCs).

A rapidly growing field of research has advanced iPSC generation and allowed for their derivation from patient fibroblasts, keratinocytes,^21^ hair follicles,^22^ peripheral blood^23^ and likely most other cell types. The viral vectors originally used for delivering the reprogramming factors resulted in potentially deleterious genomic disruptions from viral integration. Integration-free viruses, plasmids, or small molecules are now routinely used to circumvent this issue.^24^ The generation of iPSCs remains a long and labor-intensive process plagued by variability between iPSC lines.^25^ A recent approach addressed some of these issues through the use of a fully automated robotic cell reprogramming system, which may allow the generation of large cohorts of patient-specific iPSCs.^26^

One of the major advantages of the iPSC technology for modeling human diseases is that iPSCs contain the entire genetic background of the donor, making the technology particularly suitable to study diseases caused by defined genetic errors. One downside, however, is that epigenetic memory is erased during the reprogramming process. This poses a challenge to the study neuropsychiatric disorders which are greatly influenced by environmental factors known to leave their mark via epigenetic modifications. A sister technology to the traditional iPSC reprogramming method, called transdifferentiation (or direct reprogramming), circumvents the issue of epigenetic erasure by using transcription factors to directly induce a somatic cell to switch fates without passing through the pluripotent stem cell intermediate.^27^ This has been used to generate functional-induced neurons (iNs) directly from fibroblasts.^27^ Of note, transdifferentiated cells appear to retain much of their original epigenetic landscape^28^(discussed in more detail in Limitations and Moving Forward in this review). Thus, generating iNs could be a valid alternative for the modeling of environmentally induced neuropsychiatric diseases, assuming that the epigenetic changes present in the somatic cells of the patient, such as in skin fibroblasts, are relevant in the context of modeling a neuropsychiatric disease.

**Figure 2:**
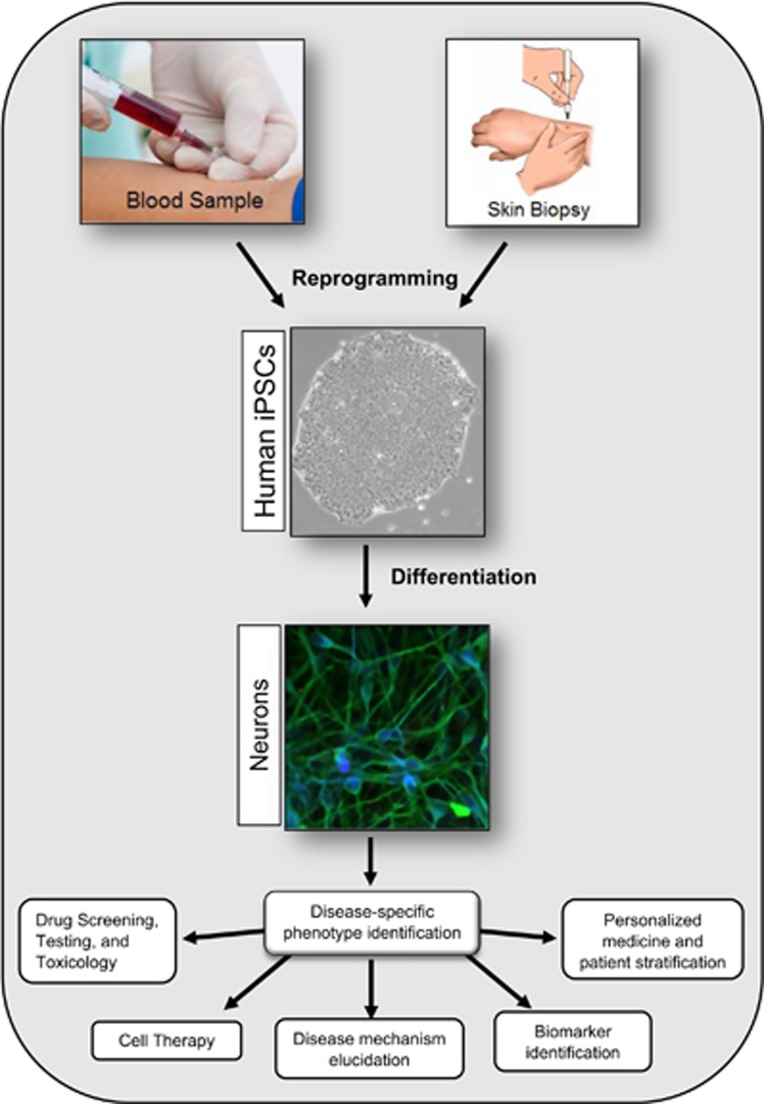
Schematic representation of induced pluripotent stem cell generation and application. A biopsy is taken from a patient (skin, blood or other tissues). Patient cells are reprogrammed into pluripotent stem cells and differentiated into the neuronal cell types of interest. Patient-derived neurons could be used for elucidating disease mechanism, high-throughput drug screening, drug testing and toxicity studies, biomarker identification and patient stratification. iPSC, human-induced pluripotent stem cell.

To date, iPSC technology has proven largely successful in modeling and recapitulating a variety of diseases *in vitro*,^29^ developing high-throughput screening (HTS) for drugs,^30, 31^ testing of new and existing drug toxicology^32^ and uncovering disease mechanisms (Figure 2).^33^ Combined with recent advances in genome editing technologies, iPSCs became an even more powerful tool. For example, the CRISPR/Cas9 genome editing tool has been used to establish causality of disease-causing mutations through their repair and introduction. Recently, progress has been made toward modeling genetically complex diseases, such as Parkinson’s disease, through the combination of CRISPR/Cas9, genome-wide association studies and epigenetics with iPSCs to address the role of a non-coding variant.^34^ In addition, CRISPR/Cas9 could be utilized to study coding variants expressed from endogenous loci and to investigate the function of single variants through isogenic controls. Hence, this technology holds promise for advancing precision medicine, patient stratification^35^ and cell-based therapies.^36^ Taken together, iPSC technology offers great potential to overcome many obstacles currently impeding progress toward understanding and treating mental illness.

## Modeling neuropsychiatric diseases using iPSCs: therapeutic implications

**Figure 3:**
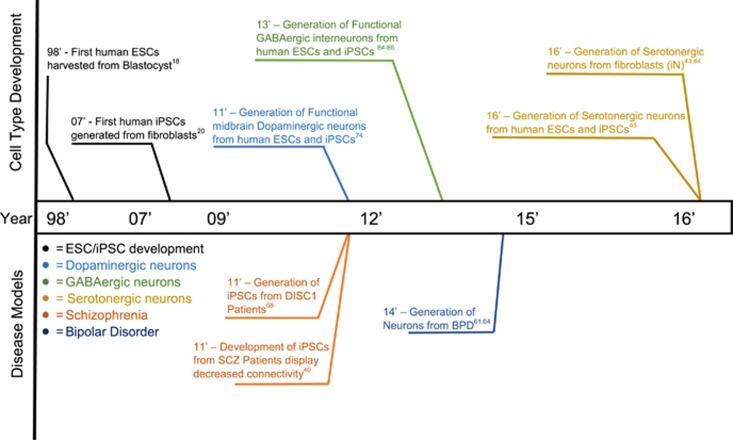
Timeline of iPSC technology development. Developments of neuropsychiatric disease models are represented on the lower side of the timeline panel, whereas developments in generating specific neuronal cell populations are represented in the upper part of the panel. BPD, bipolar disorder; iPSC, human-induced pluripotent stem cell; SCZ, schizophrenia.

Initially, researchers focused on modeling monogenetic neuropsychiatric disorders, including Rett and Fragile X syndromes^37^ (reviewed elsewhere^38, 39^). However, recent reports highlight the power of using iPSC-derived neurons to model psychiatric disorders from patients with non-monogenic causes.^40^ iPSC technology has also been used to study the role of non-coding variants associated with mental diseases.^41^ Here, we primarily highlight efforts made over the past few years to model the molecular and physiological phenotypes observed in iPSC-derived neurons from patients with the most prevalent neuropsychiatric disorders, namely depression and anxiety disorders, BPD and SCZ (Figure 3).

### Depression and anxiety disorders

MDD affects roughly 300 million people worldwide, which translates to approximately 4.5% of the global population.^42^ In 2010, it caused more DALY burden than diabetes.^42^ MDD diagnostic criteria include two or more weeks of decreased mood or markedly diminished interest or pleasure in most activities combined with one or more of the following criteria: severe change in appetite and sleeping habits, psychomotor agitation or retardation, excessive guilt, diminished concentration, or recurrent thoughts of death. First-line treatment currently includes selective serotonin reuptake inhibitors (SSRIs) and serotonin–norepinephrine reuptake inhibitors (SNRIs). Given the assumed mechanism of these medications, research has focused on finding genetic variants in the synthesis and signal conduction pathways of monoamines. Anxiety disorders follow closely behind MDD and affect about 4% of the global population.^42^ Anxiety disorders include panic, generalized anxiety, obsessive compulsive, posttraumatic stress and phobic disorders. They are all characterized by the presence of a persistent and severe anxiety, sense of dread or foreboding thoughts. First-line treatment includes a combination of psychotherapy with pharmacological agents, such as SSRIs.

Up until recently, attempts to model neuropsychiatric diseases related to serotonergic transmission *in vitro* had been unsuccessful. In 2016, two groups independently developed a method for generating serotonergic neurons via transdifferentiation directly from human fibroblasts.^43, 44^ Of note, Vadodaria and colleagues made use of citalopram, an SSRI, to show that these neurons could be potential tools for screening therapeutic compounds. In contrast, Lu *et al.*^45^ used hESCs and fibroblast-derived iPSCs to generate serotonergic neurons via temporal exposure of the cells to growth factors and modulators of signaling pathways. The group subsequently used the SSRI escitalopram to establish the utility of these neurons as screening tools for serotonin modulators. The development of reliable differentiation methods represents a leap forward in dissecting serotonin’s role in depression. If these serotonergic neurons are made from patients, they can be used to screen for new therapeutics and elucidate the unknown mechanisms through which current drugs may function.^46, 47^ This development may lead to the discovery of new drug targets and new insights into the molecular biology of depression.

Although serotonin modulators are currently first-line treatment for MDD and anxiety disorders, other neuronal populations are believed to have a role. For example, dysregulation of GABAergic neurons has also been implicated in depression and anxiety.^48^ Patients with MDD have reduced GABA receptors in the parahippocampal and lateral temporal lobes.^49^ Depressed patients showed a reduction in the number (50%) and size (18%) of GABAergic neurons in the dorsolateral prefrontal cortex and orbitofrontal cortex compared with control patients.^50^ Indeed, long-term treatment with different types of antidepressant therapies leads to enhanced GABAergic transmission through a GSK3β-mediated mechanism.^51, 52^ GABAergic cell therapy has already been applied to animal models^53, 54^ and may have a role in the treatment of MDD (for more on GABAergic based disease modeling and therapy, refer to the SCZ section of this review).

In addition, deficiencies of brain-derived neurotrophic factor (BDNF) have a role in depression, anxiety and other neuropsychiatric illnesses.^55, 56^ Indeed, the acute behavioral effects of SSRIs and tricyclic antidepressants seem to require BDNF signaling,^57^ suggesting that BDNF holds great potential as a therapeutic agent.^58^ Cell therapies focused on correcting BDNF deficiencies in mice have had some success. After a chronic stressor challenge, mice treated with human iPSC-derived neural progenitors overexpressing BDNF experienced greater neurogenesis than control mice.^59^ Thus, it is conceivable that iPSC-derived cells with inducable BDNF expression may one day be used in a form of cell-based therapy for psychiatric patients.

Collectively, the development of differentiation protocols for serotonergic and GABAergic neuronal populations will pave the way for examining the role of these populations in the pathogenesis of depression and anxiety, and may eventually open the door for cell-based therapies in human. *In vitro* models could also be used to pre-select the most effective therapy for patients with MDD and anxiety disorders, as a step towards the application of precision medicine in psychiatry.

### Bipolar disorder

BPD affects about 1% of the U.S. population.^60^ Patients with BPD present with episodes of major depression interspersed with bouts of mania or hypomania. First-line treatment includes mood stabilizers, such as lithium and valproic acid. Over the past 2 years, a few groups managed to generate iPSC-derived neurons from fibroblasts isolated from patients with BPD and healthy control patients.^61, 62, 63, 64^ In one study, iPSC lines were derived from two brothers with BPD and their unaffected parents.^62^ Several genes were differentially expressed in BPD-derived neuronal precursor cells, most of which regulate neuronal differentiation, projection and calcium binding. Interestingly, neuronal precursor cells generated from BPD patients showed impaired neural differentiation and decreased proliferation, both of which were rescued by treatment with a selective inhibitor of the enzyme GSK3β (a known target of lithium therapy).^62^ Another group reprogrammed fibroblasts derived from patients with BPD, half of whom were responsive to lithium treatment, to iPSC-derived hippocampal dentate gyrus granule cell-like neurons, which are reportedly affected in BPD.^63^ Of note, BPD-derived neurons showed altered expression of mitochondrial, calcium-signaling and neuronal excitability genes. In addition, when compared with controls, BPD-derived neurons exhibited a hyperexcitability phenotype as evidenced by higher frequency of spontaneous action potentials. Remarkably, a 1-week treatment with lithium partially normalized changes in mitochondrial gene expression and hyperexcitability phenotype only in neurons derived from patients with BPD who were responsive to lithium.^63^ These recent findings suggest a role for mitochondrial signaling in BPD and shed light on potential molecular mechanisms that could explain the differences in patient responsiveness to lithium treatment.^65^

Seeing a specific phenotype in disease-relevant neurons *in vitro* represents an exciting first step in the development of an iPSC-based disease model. However, it is often challenging to determine the significance of an *in vitro* phenotype for a given disease. Such phenotypes are difficult to evaluate given the lack of primary tissue from patients for further confirmation and the overall lack of understanding of disease-initiating events in neuropsychiatric diseases. One desirable endpoint is to use such disease-related phenotypes as the starting point for HTS, which allows for testing hundreds of compounds simultaneously. One group has developed a HTS for testing various compounds on human iPSC-derived neurons for modulators of the Wnt/GSK3β signaling system,^66^ a system further validated through the use of lithium.

### Schizophrenia

SCZ affects more than 21 million patients worldwide.^67^ Diagnosis is made when a patient presents with at least 6 months of perturbed language, perception, thinking, social activity, affect and volition. First-line treatments include atypical antipsychotics, such as olanzapine and risperidone. SCZ was among the first neuropsychiatric disorders modeled with patient iPSC-derived neurons.^40, 68^ In one of those studies, SCZ-iPSC-derived neurons showed decreased connectivity, synapses, spine density and expression of glutamate receptors when co-cultured with human astrocytes.^40^ Interestingly, treatment with the dopaminergic antagonist loxapine, but not clozapine, olanzapine, risperidone or thioridazine, during the last 3 weeks of neuronal differentiation increased neuronal connectivity in iPSC-derived neurons from all patients. The inability of the structurally similar antipsychotic clozapine to rescue phenotypes observed in defective neurons raises the question about the exact mechanism by which loxapine acts in this system. Another group generated iPSC lines from family members carrying mutant *DISC1* and demonstrated synaptic defects in those iPSC-derived neurons.^33^ Genes related to synaptic transmission and development were dysregulated, about 90 of which had been previously linked to mental disorders, including depression, BPD and SCZ. Intriguingly, using gene editing techniques, the group established a causal link between the *DISC1* mutation and the observed defects.^33^

One of the mainstream theories behind the pathogenesis of SCZ is dopamine dysregulation. Studies supporting this theory show aberrant dopamine transmission within multiple brain regions,^69^ including the amygdala,^70^ prefrontal cortex^71, 72^ and hippocampus.^73^ Our group has established an efficient differentiation protocol for generating midbrain dopaminergic neurons, which showed long-term survival when engrafted in mouse, rat and monkey models.^74^ Numerous groups have used and adapted this protocol for the generation of dopaminergic neurons from patients with SCZ.^75, 76^ Dopaminergic neurons differentiated from iPSCs derived from patients with SCZ had a reduced neurite count and dopamine release and showed delayed maturation.^76^ Interestingly, these defective neurons exhibited perturbations in mitochondrial network structure and connectivity. This suggests a potential mitochondrial defect having a key role in the pathogenesis of SCZ, much like that reported in BPD.^63^ Reports indicating that iPSCs from some patients with SCZ show poor differentiation into dopaminergic neurons,^75^ suggesting a need to improve current protocols before those neurons can serve as a robust platform to study the factors contributing to the dysregulation of dopamine in SCZ. Such further improvements may also facilitate the development of cell-based screens for the identification of novel candidate therapeutic compounds or to guide future treatment choices in individual patients.

Studies have tied the hyperfunctioning of the dopaminergic system to hyperexcitability in the hippocampus,^77, 78^ one of the potential drivers of which is the reduced number of GABAergic interneurons providing tonic inhibitory control.^79, 80^ Human studies also suggest a major role for GABAergic dysfunction in SCZ. Post-mortem studies of SCZ brains reveals a 40% reduction in GABAergic synapses,^81^ which is a deficiency that may originate during development.^82^ Indeed, grafting GABAergic neurons into the ventral hippocampus in the rodent model of SCZ restored normal hippocampal functioning, corrected downstream dopaminergic dysregulation and rescued behavioral deficits.^54, 83^

To better probe the nature of GABAergic interneurons and their potential role in disease pathogenesis, several groups have developed differentiation protocols to produce these neurons using hESCs or iPSCs.^84, 85, 86^ Interestingly, these studies provide evidence that human GABAergic interneurons have a lengthy maturation period, requiring as long as 7 months to reach mature phenotypes. The ability to generate interneurons opens the door to potential GABAergic cell-based therapies in the treatment of SCZ^87^ and other diseases associated with GABAergic dysfunction, such as depression,^50^ epilepsy^53^ and neuropathic pain.^88^

## Limitations and moving forward

Human iPSC models provide an invaluable tool to dissect the molecular and pathophysiological defects underlying neuropsychiatric disorders and offer a powerful platform for drug screening in disease-relevant cells. Although this system addresses many challenges associated with studying mental illness, important limitations remain.^89^ First, epigenetic erasure: the current dogma in the field states that upon reprogramming, not only is the cellular lineage identity reset, but the epigenetic landscape of the cell is erased.^90^ This gives the cell a relatively clean slate on which to form the modifications necessary to differentiate into any cell type of the body, much like the germline reprogramming that facilitates totipotency in the mammalian zygote.^91^ However, this is problematic for the study of neuropsychiatric diseases, such as depression or anxiety, which are greatly influenced by environmental factors known to modify epigenetics. There is evidence that some epigenetic memory is retained through the reprogramming process, which is supported by the tendency of iPSCs derived from blood cells to preferentially differentiate toward mesoderm lineages (that is, their lineage of origin).^28^ However, such epigenetic memory may not persist long-term in iPSCs as repeated passaging at the pluripotent state is correlated with a loss of the lineage-specific differentiation bias.^92^ Furthermore, there is a possibility that some retained epigenetic memory represents incomplete reprogramming of the iPSCs. Transdifferentiated cells (iNs) appear to retain much of their original epigenetic landscape.^93^ Thus, generating iNs could be a valid alternative for the modeling of environmentally induced neuropsychiatric diseases. Although induced neuron technology may better preserve the epigenetic landscape of the patient donor cell, epigenetic modifications vary across different cell types and therefore changes in skin fibroblasts may not capture important neuron-specific disease-related epigenetic differences.^94^ The emerging field of transgenerational epigenetic inheritance provides evidence that environmental influences, including nutrition or stress, can modify epigenetic marks inherited across the germline.^95, 96^ Thus, such marks may persist through the reprogramming process and be of use in the study of mental illness with environmental contributions.

Second, developmental state: neurons differentiated from iPSCs most closely resemble cells of the fetal brain, a problem for modeling diseases that emerge later in life.^97^ This topic is the subject of active research aiming at developing techniques to mimic cellular maturation and aging.^98^ Third, current protocols for generating iPSCs vary in efficiency and produce heterogeneous populations.^25^ This could be due to the fact that iPSCs accumulate mutations in culture over time.^99^ Although transdifferentiation techniques skip potentially crucial phases of neuronal development, these techniques offer a potential solution for this problem.^100^ Fourth, current techniques for differentiating iPSCs yield a heterogeneous population of neuronal subtypes, which may have varying roles in disease pathogenesis.^101^ Future protocols differentiating iPSC into more precisely defined neural subtypes may reveal phenotypes that are hidden using current protocols. Fifth, the expensive and time-consuming nature of generating iPSCs and differentiating them into neurons often leads to small sample sizes for most reported studies. Currently, scaling up the experiments to include more subjects remains a major obstacle. However, new techniques are being developed to reduce the time and costs required to generate patient-specific neurons.^101^ Sixth, the pathogenesis of many neuropsychiatric diseases most likely lies at the level of neuronal circuits, making them difficult to study using iPSC technology. Co-culture systems^102^ and 3-dimensional organoids^103, 104, 105^ provide systems potentially capable of recapitulating circuit level interactions, to address this challenge. Importantly, iPSC-derived neurons have also been grafted into animal models to observe their circuit level interactions *in vivo*.^74, 106, 107, 108^

## Conclusions

Neuropsychiatric disorders affect a large proportion of the global population, and continues to affect more people every year. There is a tremendous need to identify pathophysiology of these disorders and find novel, targeted treatments. Major limitations in neuropsychiatric research include the lack of access to disease-specific cell types. iPSC technology offers new opportunities to model disease-relevant neural cells from patients. Over the past 5 years, a handful of groups have applied this technology toward studying mental disorders, including SCZ and BPD (Table 1). Remarkably, these groups have developed differentiation protocols capable of generating specific cell populations, such as GABAergic, serotonergic and dopaminergic neurons. Despite the exciting progress made in recent years, this represents only a fraction of the potential iPSC technology holds for unlocking insights into the cause of mental illness. Although our knowledge of the causes behind mental illness has lagged, iPSC technology will push boundaries in our ability to understand, diagnose and treat neuropsychiatric disorders.

### Search strategy and selection criteria

Data sources were identified through searches in PubMed. Search terms were as follows: (pluripotent OR transdifferentiation) AND (psychiatr* OR schizophrenia OR bipolar OR depressi* OR Anxiety). This yielded 374 articles as of June 2016. Publications in English were used.

## Figures and Tables

**Table 1:** Selected human iPSC-based reports modeling SCZ and BPD

*Disease*	*Authors, year, journal*	*Neuronal cell*	*Main finding*
SCZ	Chiang *et al.*, 2011, *Mol Psychiatry*^68^	NA	This study presents the first iPSC lines derived from SCZ patients.
	Brennand *et al.*, 2011, *Nature*^40^	NPCs and neurons	Decreased connectivity, neurite number, diminished cAMP, Wnt signaling and levels of PSD95 protein in SCZ-derived neurons. Defective phenotype was reversed by the antipsychotic Loxapine
	Pedrosa *et al.*, 2011, *J Neurogenetics*^109^	Glutamatergic neurons	First study to model the SCZ risk factor 22q11 using patient-derived iPSCs.
	Robicsek *et al.*, 2013, *Mol Psychiatry*^76^	NPCs, dopaminergic and glutamatergic neurons	Differentiation and maturation deficiencies, and mitochondrial defects in patient-derived neurons
	Hook *et al.*, 2014, *Stem Cell Reports*^110^	NPCs and neurons	Increased secretion of catecholamines, higher numbers of TH^+^ neurons in patient-derived neurons
	Siegert *et al.*, 2015, *Nat Neuroscience*^111^	Direct conversion of fibroblasts to neurons	Increasing miR-137 expression caused downregulation of presynaptic target genes, impaired vesicle release, impaired synaptic plasticity in hippocampus. These phenotypes were rescued by sequestering miR-137
	Srikanth *et al.*, 2015, *Cell Rep*^112^	NPCs and neurons	Increased level of canonical Wnt signaling, and altered expression of neuronal fate markers such as Foxg1 and Tbr2 in NPCs derived from DISC1-mutated cells. Gene expression changes are rescued by antagonizing Wnt signaling in a critical developmental window
BPD	Chen *et al.*, 2014, *Transl Psychiatry*^61^	Mixed glutamatergic-GABAergic Neurons	Increased expression of membrane-bound receptors and ion channels in BPD-derived neurons. Exposure of BP neurons to lithium produced significant alterations in wave amplitude and calcium transient.
	Wang *et al.*, 2014, *Transl Psychiatry*^64^	Neurons (transdifferentiation)	Cell adhesion was associated with clinical response to lithium treatment
	Bavamian *et al.*, 2015, *Mol Psychiatry*^113^	NPCs and neurons	miR-34a levels are increased in BPD patient-derived neuronal cultures. Reducing endogenous miR-34a expression enhances dendritic elaboration.
	Madison *et al.*, 2015, *Mol Psychiatry*^62^	NPCs	Abnormalities in early steps in NPC formation, WNT/GSK3 signaling and ion channels expression in the BPD patient-derived NPCs and neurons. Rescue of proliferation defects in BPD patient NPCs with GSK3 inhibition
	Mertens *et al.*, 2015, *Nature*^63^	Hippocampal dentate gyrus-like neurons	Changes in the expression of genes linked to mitochondrial function and neuronal excitability; reduced mitochondrial size and enhanced function in BPD neurons. Lithium reduced the hyperexcitability of LR neurons and partly normalized their mitochondrial function

Abbreviations: BPD, bipolar disorder; iPSC, induced pluripotent stem cells; NA, not available; NPC, neural progenitor cells; SCZ, schizophrenia.
